# A simple flash and freeze system for cryogenic time-resolved electron microscopy

**DOI:** 10.3389/fmolb.2023.1129225

**Published:** 2023-03-07

**Authors:** Biddut Bhattacharjee, Md Mahfuzur Rahman, Ryan E. Hibbs, Michael H. B. Stowell

**Affiliations:** ^1^ University of Colorado Boulder, Boulder, United States; ^2^ University of Texas Southwestern Medical Center, Dallas, United States

**Keywords:** CryoEM, time resolved, LED, optogenetic activation, caged compounds, plunge freezer, plug & play, vitrification

## Abstract

As the resolution revolution in CryoEM expands to encompass all manner of macromolecular complexes, an important new frontier is the implementation of cryogenic time resolved EM (cryoTREM). Biological macromolecular complexes are dynamic systems that undergo conformational changes on timescales from microseconds to minutes. Understanding the dynamic nature of biological changes is critical to understanding function. To realize the full potential of CryoEM, time resolved methods will be integral in coupling static structures to dynamic functions. Here, we present an LED-based photo-flash system as a core part of the sample preparation phase in CryoTREM. The plug-and-play system has a wide range of operational parameters, is low cost and ensures uniform irradiation and minimal heating of the sample prior to plunge freezing. The complete design including electronics and optics, manufacturing, control strategies and operating procedures are discussed for the Thermo Scientific™ Vitrobot and Leica™ EM GP2 plunge freezers. Possible adverse heating effects on the biological sample are also addressed through theoretical as well as experimental studies.

## Introduction

**FIGURE 1:**
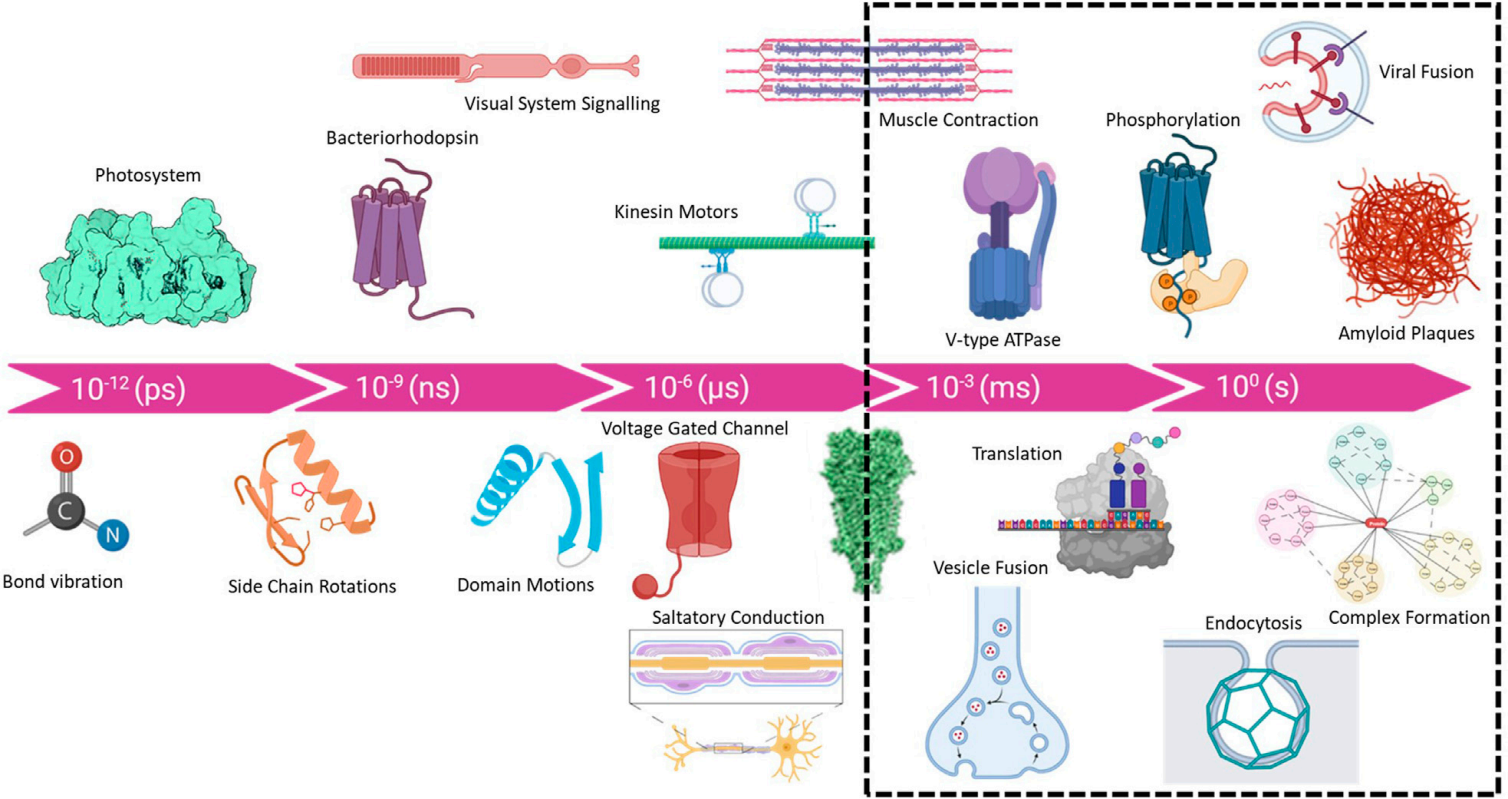
Timescale of biological processes from bond vibrations and charge separation (femto and pico second) to complex assembly (minutes to hours) and even amyloid plaque formation in the human brain (years). Although new methods are being developed, the practical range for cryoTREM is limited by the rate of sample cooling which is ∼10,000 K/s and places a lower limit of ∼1 ms due to the cooling rate using traditional plunge freezing instruments.

Macromolecular complexes are flexible dynamic systems that undergo a myriad of structural changes in response to all manner of perturbations. The kinetics and magnitudes of these structural changes cover a broad range from picoseconds to years and bond-vibrations to protein aggregation and plaque formation Figure 1. The structural investigation of intermediate states provides crucial information for mapping the reaction coordinates of biological systems and helps to elucidate mechanisms of action. Accessing such intermediate states has historically been achieved using pharmacological agents and/or site-specific mutagenesis to “trap” intermediates states. These methods have provided important insight into many biological systems, e.g., the use of non-hydrolysable ATP analogs to trap ATPases in the apparent transition state. The use of active site mutants has similarly expanded our knowledge of intermediate states of various enzymes and molecular machines. Non-etheless, many biological complexes are not amenable to such methods or multiple intermediate states exist that cannot be readily trapped through chemical or mutational means. Additionally, the ability to directly determine native intermediate structural states in the absence of mutagenesis and or inhibitor trapping would provide more rigorous and reliable data for mapping structural transitions. Substantial efforts have pursued the use of ultrafast X-ray lasers to study charge separation in photosystems (Kupitz et al., 2014) and the use of Laue methods in time-resolved X-ray crystallographic studies has a long and storied history (Moffat, 2019). Such methods are suitable for very rapid processes (<1 ns), with minor conformational changes that are not expected to be affected by crystal contacts. Conversely, cryoEM is ideally suited for studying large conformational changes on the timescale of milliseconds to minutes. Furthermore, as modern computational approaches in cryoEM can 3D classify particles, branched conformational change pathways can be quantified and resolved. While the practical lower limit of 1 ms is governed by the cooling rate of the plunge freezing method (Dubochet, 2007; Frank, 2017), *in-situ* rapid heating and cooling methods may provide access to microsecond timescales (Voss et al., 2021; Bongiovanni et al., 2022).

Time-resolved studies have been at the heart of fundamental understandings of many biological complexes including enzymes, channels, photosystems, and molecular motors. Early efforts in cryoTREM began with a series of foundational studies on the nicotinic acetylcholine receptor using a spraying method to capture the open state of the acetylcholine receptor (Berriman and Unwin, 1994; Unwin, 1995). Helical crystal samples were sprayed just prior to plunge freezing in liquid ethane to capture the open state of the receptor and included ferritin particles as fiducials to identify regions where droplets had mixed with the sample. Subsequent efforts by several groups have adopted similar strategies using a variety of similar methods including on the fly droplet mixing and pre spray microfluidic mixing (White et al., 2003; 1998; Lu et al., 2014; Chen et al., 2015; Feng et al., 2017; Kaledhonkar et al., 2018; Fu et al., 2019; Dandey et al., 2020). The main disadvantages of the system are the requirement for specialized equipment and most importantly the control of the liquid film thickness prior to plunge-freezing. Film thickness in particular is critical factor in obtaining quality data and is routinely measured during data collection (Rice et al., 2018). This ensures that the film is thin enough for rapid cooling but not too thin as to damage particles due to the air-water interface.

Alternatively, the use of light to trigger biological activity is well-established in the field of rapid enzyme kinetics and was first successfully implemented in cryoEM to investigate conformational changes in bacteriorhodopsin (Subramaniam et al., 1999; Subramaniam et al., 1993; Subramaniam and Henderson, 1999) and an automated device for flash-photolysis and flash-freezing was subsequently described (Shaikh et al., 2009). Despite these early successes and developments in the use of light activation for cryoTREM this approach has not seen substantial adoption. This is surprising considering the many advances in the development of caged compounds and optogenetic tools which have been implemented with great success in many fields many of which have become commercial products (Tischer and Weiner, 2014; Ferenczi et al., 2019; Ellis-Davies, 2020; Monteiro et al., 2021). One of the major concerns with the use of light for cryoTREM is the potential for thermal heating of the sample due to absorption by both the sample and the EM grid. Several early studies suggested that such heating would preclude the use of light for all but the most quantum efficient systems such as bacteriorhodopsin (Siegel et al., 1994; Shaikh et al., 2009). However, these studies utilized broad band light sources and temperature measurement methods that did not control for the photoelectric effect. In fact, recent work has demonstrated minimal apparent heating in plunge frozen samples irradiated with a narrow band high-power LED (Yoder et al., 2020).

Here we provide detailed analysis of the potential use of light as a trigger for cryoTREM systems. Using both theoretical and experimental studies, we determine the optimal parameters for a light triggered cryoTREM system employing low-cost LED light sources suitable for exploiting a wide range of readily available caged compounds and optogenetic tools. These data were then used to guide the design, building, and testing of a low-cost LED light source suitable for plug and play into the two most widely used automated plunge-freezers, the Thermo Scientific Vitrobot Mark IV and the Leica EM GP2. We provide a comprehensive analysis of light absorption and thermal heating of copper and gold EM grids and measure temperature jumps for several wavelengths and irradiation times on the order of milliseconds. A description of the components is provided so that others can readily construct such instrumentation for future cryoTREM studies.

## Results

The success in light activated uncaging of compounds to capture the desired intermediate states of samples of interest will depend on many factors. The required irradiation power is one of the basic factors that must be known or estimated. Table 1 lists the calculated power requirement for some commonly available caged compounds and photoactive proteins. For these calculations we assumed an exposure of 20 milliseconds, 100 nm liquid depth after blotting, the concentration of the caged compound 10 mM, and the desired concentration of the released product 200 µM.

**TABLE 1:** Exemplary photoactive compounds/proteins and associated photophysical properties.

Compound/System	ε (M^−1^cm^−1^)	Peak abs (nm)	Quantum yield	Required power (mW/cm^2^)
NPE caged Proton (1-(2-Nitrophenyl)ethyl sulfate sodium)	3000	335	0.47	104
N-[(α-carboxy)-2-nitrobenzyl] carbamoylcholine	500	350	0.8	371
DMNPE ATP	5300	354	0.63	44
DM-nitrophenyl Calcium	4330	350	0.18	191
4-methoxy-7-nitroindolinyl (MNI) Glutamate	4820	336	0.085	378
Channel Rhodopsin	50000	480	0.67	3.6
Bacteriorhodopsin	63000	568	0.64	2.3
Cryptochrome, Cry2	5000	380	0.213	128
PhoCl photocleavable protein	34000	488	0.73	4.3
Azobenzene (trans-cis)	21000	310	0.11	73
3′,5′-bis(carboxymethoxy)benzoin	252	320	0.64	1010

One of the major concerns in the light activation of caged compounds or optogenetics samples on a copper or gold grid is the thermal effects due to radiation absorption. The absorption coefficient (optical depth, α) of water in the 300–700 nm range is between 0.01 m^−1^ and 0.1 m^−1^. Considering the typical aqueous layer thickness of 100 nm after blotting, the temperature rise due to a 1 W/cm^2^ beam of 365 nm radiation for 1 s is estimated to be 0.0034°C ( $∆T=P_{i}At\left(1-e^{-\alpha d}\right)/mC_{p}$ , where *P*_*i*_: incident power per unit area (Irradiance), *A*: Grid area, *t*: exposure time, *d*: water depth, *m*: mass of water, *C*_*p*_: heat capacity). Therefore, the absorption by water can be safely neglected. However, metals interact with radiation to varying degrees depending on the frequency and the type of metal.

The system we report here is designed for light sources of wavelengths 365 nm and 455 nm. The following discussion will be based on 365 nm radiation. Due to the finite conductivity of a given metal, the incident electromagnetic wave is attenuated to zero intensity within a certain distance from the surface. This distance is characterized by the ‘skin/penetration depth’ or ‘attenuation depth’ given by $\delta =\sqrt{2/\mu \sigma \omega }$ Where, *µ*: permeability of the metal, *σ*: electrical conductivity, *ω*: frequency of light. This characterizes the depth of metal within which the incident electromagnetic wave attenuates to essentially zero magnitude. The origin of this attenuation is the resistive dissipation of energy. Table 2 shows the skin depth for noble metals copper and gold for two different wavelengths. Importantly, although these values indicate that 365 nm and 450 nm will be completely blocked by metal layers of ∼5*δ* (∼15 nm), it is well-known that noble metal films of much greater thicknesses are used as absorbance standards to partially attenuate light (Axelevitch et al., 2012; Son et al., 2015). The power dissipated as a percentage of the incident power is given by $\frac{P_{d}}{P_{i}}=\sqrt{\frac{8\omega \varepsilon_{0}}{\sigma }}$ , where ε_0_ is the permittivity of vacuum. Values for copper and gold are shown in Table 3 for two wavelengths. Again, experimental values of reflectivity ( $R=1-\frac{P_{d}}{P_{i}}$ ) of gold and copper at the above wavelengths are significantly different (Johnson and Christy, 1972; Babar and Weaver, 2015). For instance, copper and gold absorb about 58% and 65% of the electromagnetic wave of wavelength 365 nm, respectively.

**TABLE 2:** Skin depth penetration calculations.

	Skin depth, nm		P_d_/P_i_	
	365 nm	450 nm	365 nm	450 nm
Copper	2.3	2.6	8.0%	7.1%
Gold	2.7	3.0	9.4%	8.5%

**TABLE 3:** Finite conductivities of copper and gold.

	% reflectivity (365 nm, 3.4 eV)	% reflectivity (450 nm, 2.75 eV)	Plasma frequency (X 10^15^ Hz), [wavelength, nm]	Collision frequency (X 10^12^ Hz), [wavelength, µm]	Fermi energy (E_F_), eV	Valence *d*-band (E_F_-E_d_), eV	Conductivity, (S/m)	Heat capacity, J/Kg.^o^C	Density, Kg/m^3^
Copper	42	54	2.614, [115]	6.55, [45.7]	7	2	5.8 × 10^7^	377	8960
Gold	36	37	2.178, [138]	6.42, [46.7]	5.5	2.45	4.1 × 10^7^	129	19300

The above simplified models are based on very high electrical conductivities, i.e., $\sigma \gg \omega \varepsilon_{0}$ . However, the finite conductivity of metals is the consequence of collision of charge carriers. In copper the collision frequency of electrons is estimated to be 6.55 THz, corresponding to infrared radiation of wavelength 46 μm, as shown in Table 3. Thus, the conductivity of copper (5.8 × 10^7^ S/m) will remain the same for EM waves of frequencies much lower than the collision frequency. In the near infrared and visible range, the DC conductivity of copper does not provide accurate values. In this high-frequency regime one needs to take the reactive part of the conductivity into account.

The free-electron theory of metals attempts to explain the behavior of metals when the EM wave frequency is much greater than the collision frequency, i.e., the near-infrared and visible spectra. Due to the higher frequencies, electrons in metal are collectively treated as a plasma gas, and the behavior of metals are characterized by the plasma frequency. This frequency is defined as, $\omega_{p}=\sqrt{\frac{N{q_{e}}^{2}}{m_{e}\varepsilon }}$ , where is N is the number of electrons per unit volume, $q_{e}$ is electron charge, $m_{e}$ is electron mass, and ε is permittivity. The effective permittivity of metals is given by $\varepsilon_{eff}=\varepsilon_{0}\left(1-\frac{{\omega_{p}}^{2}}{\omega^{2}}\right)$ . When the incoming electromagnetic wave has a frequency higher than $\omega_{p}$ , the permittivity becomes positive and the wave propagates through the metal. Below $\omega_{p}$ , the permittivity is negative, and metals are supposed to reflect the incoming wave. As shown in Table 3, the values of $\omega_{p}$ for copper and gold correspond to wavelengths of 115 nm and 138 nm, respectively. However, experiments show that copper and gold films do absorb starting from wavelengths of around 590 nm and 520 nm, respectively, to lower wavelengths (Staelin et al., 1994).

The electronic band structure of noble metals helps greatly to understand the optical behavior. Copper and gold have bound electrons in the completely filled valence *d*-bands (3*d* and 5*d*) below the Fermi energy (*E*_*F*_) level. Electrons in the s-band can be continuously excited by low frequency EM waves. However, electrons in the *d*-band can be excited above *E*_*F*_ when photons of frequency $\omega =2\pi \left(E_{F}-E_{d}\right)/h$ interact with them (Wooten, 2013). This means photons of frequency 508 THz (590 nm, 2.1 eV) will impart energy to bound electrons in the 3*d* band of copper to occupy unfilled states above the Fermi level. Thus both the free electrons in the *s* band and the bound electrons in the *d* band contribute to the optical behavior of noble metals. The total dielectric function then consists of a free-electron part and a bound-electron part: $\varepsilon =\varepsilon_{0}\varepsilon^{f}+\varepsilon_{0}\varepsilon^{b}$ , where $\varepsilon^{f}$ is the dielectric function due to conduction (free) electrons, and $\varepsilon^{b}$ is that due to bound electrons (Ehrenreich and Philipp, 1962). It must be noted that both are complex quantities consisting of a real and an imaginary part. The absolute value of the imaginary part is related to the absorption of energy. Therefore, it is convenient to express the dielectric function as follows: $\varepsilon =\varepsilon_{0}\varepsilon_{r}+i\varepsilon_{0}\varepsilon_{i}$ , where $i^{2}=-1$ . The positive contribution of bound electrons results in less negative values of the real part of the dielectric constant, and ultimately making it positive for some metals at frequencies which are lower than those predicted by the theoretical plasma frequency relation.

The wave number or the propagation constant in metals is given by $k=\frac{\omega }{c}\sqrt{\varepsilon_{r}+i\varepsilon_{i}}=k_{r}+k_{i}$ , where the real part determines the wavelength inside the metal and the imaginary part signifies damping of the wave. Hence, the EM wave inside a metal is oscillation in fields whose amplitude decreases exponentially with the distance travelled. The distance from the surface of metal by which the amplitude is reduced to 37% (= *e* ^*−1*^) is called the penetration depth, $D_{p}=1/k_{i}$ . Using the experimentally measured values of the dielectric constants, we find the penetration depth of 365 nm light in copper and gold to be 28 nm and 33 nm, respectively. These values are significantly greater than those shown in Table 1. This explains why up to 80 nm thin films of gold partially transmits (∼70%–20%) radiation of wavelengths between 300 and 530 nm (Axelevitch et al., 2012; Son et al., 2015).

The rise in temperature can be estimated from the properties of the grid and the irradiance of the exciting source. The dissipative power per unit area as a fraction of the total irradiance is given by:

$$\frac{P_{d}}{P_{i}}=1-{\left|\Gamma \right|}^{2}$$

where $P_{d}$ is the power dissipated per unit area of the grid (W/m^2^), $P_{i}$ is the irradiance (W/m^2^), $\Gamma =\frac{1-{\sigma D}_{p}\eta_{0}}{1+{\sigma D}_{p}\eta_{0}}$ is the complex reflection coefficient, and ${\left|\Gamma \right|}^{2}$ is the reflectivity. $\eta_{0}$ is the vacuum impedance. Using the above model, we find the absorbed power for copper and gold to be 62% and 69% of the incident power with 365 nm light. We note that the aim of our simplified theoretical effort is to understand qualitatively the sources and mechanisms of light absorption near the green, blue and UV wavelengths, and the consequent temperature rise of the commonly used grid materials. The full description of the light-metal interaction is possible, however, only through quantum-mechanical analysis. It is important to note that the conductivity, σ , used here is not the DC conductivity, but a complex number whose components are defined by the components of the complex dielectric function. Whereas the DC conductivity values of noble metals are high and real, the complex conductivity has a frequency-dependent real part whose magnitude may be significantly lower than the DC value. As a result, the dissipative loss manifested as heating the metal is higher in the optical frequencies. Moreover, the frequency-dependent imaginary part of the conductivity contributes to the stored energy. Overall, the frequency-dependent conductivity accounts for the higher absorbance of shorter wavelength light by the common grid material.

**FIGURE 2:**
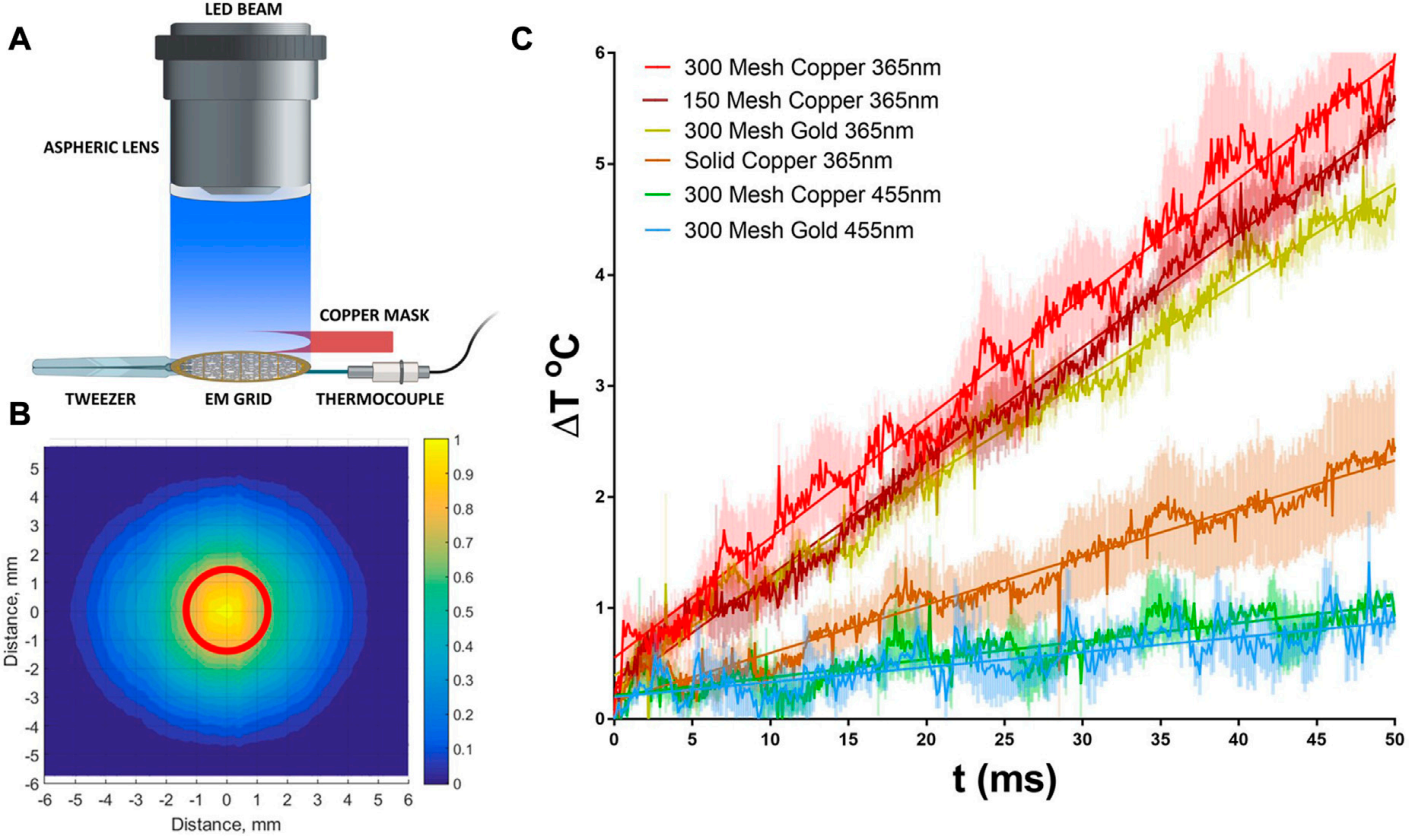
Measurement of thermal heating by UV (365 nm) and Visible (455 nm) LED beam. **(A)** Experimental setup that replicates the experimental conditions within the Vitrobot during light triggering, see methods. **(B)** Measured beam profile demonstrating the beam uniformity within the EM grid diameter (red circle). 2D intensity profile was constructed from a series of signals measured by a linear photodiode array, see methods. **(C)** Temperature rise curves during irradiation for several grid types typically used in cryoEM at both 365 and 455 nm. Data are averages of 3 separate measurements using the measurement setup in A and errors are shown as shaded color.

To complement these theoretical studies, we constructed a thermal test bed for both copper and gold grids that can provide a direct experimental measurement of temperature rise due to irradiation. The system is comprised of a liquid light guided LED, coupled to an aspheric lens to provide uniform beam output, Figures 2A, B. The use of an aspheric lens to achieve beam uniformity is critical for both these measurements as well as future implementation in cryoTREM to ensure that all regions of the sample are activated equally and minimizes off axis translation variation in beam intensity as occurs with a typical focusing lens (Yoder et al., 2020). Using this test bed, we measured the temperature rise as a function of time for a 2 W/cm^2^ 365 LED beam, Figure 2C. The data demonstrate a minimal temperature rise for the targeted power density and irradiation time with nominal variation between grid types and excellent agreement with the above theoretical analysis.

Both copper and gold are excellent conductors of heat, and the thermal diffusivities are ∼118 × 10^6^ μm^2^/s and ∼126 × 10^6^ μm^2^/s respectively. For grids of thicknesses 25 μm, the estimated timescale for the temperature gradient between the top and the bottom surfaces to subside are in microseconds. Hence, the readings from the thermocouple from the bottom surface are expected to be accurate representation of the instantaneous bulk temperature of the grid. Figure 2 shows the temperature rise of different types of grid when subject to the 365 nm light beam. The grid temperature increases linearly with time due to the absence of any significant heat loss during the early stage of irradiation. However, this rise in temperature slows down after about 80 ms (data not shown) mainly due to heat transfer to the tweezer.

The reliability of temperature data can be compared to the theoretical values applying the fundamental relation $\frac{∆T}{Q}=\frac{1}{\rho hAC_{p}}$ , where *Q* is the radiation energy (J) absorbed by the grid surface, *ρ* is the density (Kg/m^3^), *h* is grid thickness (m), *A* is the area (m^2^) of the grid, *C*_*p*_ is the heat capacity (J/Kg.^o^C). If we assume that all the incident energy is absorbed, then for a 300 mesh copper grid of thickness 25 µm we find a value of 1.67°C/mJ. This is the maximum value derived from fundamental physics. As shown in Figure 2, the measured temperature rise per unit of radiation energy applied to this grid is 1.07°C/mJ. This implies that a copper grid absorbs about 64% of the energy of a 365 nm beam, which is in agreement with published absorptivity value of ∼58% (Ehrenreich and Philipp, 1962; Johnson and Christy, 1972; Babar and Weaver, 2015).

It is noted that the surface quality of metals affects light absorption considerably (Gevorkian et al., 2022). We used commercially available grids without any surface modification and the deviation of the estimated absorptivity calculated from the measured temperature rise versus the published values might be due to the presence of rough peaks and valleys, the non-polarized character of the beam, and the mesh structure. The measured temperature rise per unit of irradiation energy for gold grids is 0.86°C/mJ. This is less than the theoretical value relative to that of copper grids as found from $\frac{∆T^{g}}{∆T^{c}}=\frac{a^{g}\rho^{c}C_{p}^{c}}{a^{c}\rho^{g}C_{p}^{g}}$ , where superscripts *g* and *c* stands for gold and copper, respectively, and *a* is the absorptivity at a given wavelength. Thus, the temperature rise in gold grids is expected to be 1.52 and 1.92 times that of copper grids at 365 nm and 450 nm, respectively. As mentioned above, the smaller change of temperature in gold might be due to the more polished surface relative to copper grids. The simulation studies for both grids show an approximately linear rise due to the absence of significant convective and radiative losses. However, the rate of change of temperature slows gradually due to the tweezer acting as a large heat sink.

In summary, the measured temperature rise of grids in our system is well within the theoretical limit, and reasonably close to the published absorptivity values for mirror finish metal thin films. However, we want to emphasize here that the photothermal heating of grids is significantly overestimated in some publications (Siegel et al., 1994; Shaikh et al., 2009). Siegel et al. (1994) reported a temperature rise of 51°C in 1 ms in a copper grid subject to a Xenon flashtub. This corresponds to about 430 W/cm^2^ light power incident on the grid and fully absorbed. While it is possible to generate and apply this level of power, a lower irradiance is preferred to avoid any undesired biological activity. Surprisingly, these early studies using a xenon lamp-based flash system reports grid temperature rises of ∼43°C in 5 ms, which translates to ∼3.2°C/mJ after taking the applied power into account (Shaikh et al., 2009). This value is almost twice the theoretical limit of 1.67°C/mJ. These previously reported temperature rises due to irradiation are overestimations originating from the measurement methods. In both studies, wires of copper and copper-nickel alloy were either woven into or spot-welded onto copper grids. Nickel absorbs all frequencies of radiation since it has a partially occupied *d*-band. Therefore, exposing the thermocouple joints to strong radiation, particularly in the 350–400 nm range, results in a photoelectric effects that gives rise to a spurious response of the thermocouple.

## Conclusion

Here we have combined theoretical and experimental studies to demonstrate that light activated cryoTREM is feasible with nominal heating of the sample due to EM grid absorption. Importantly, the theoretical and experimental results reported here are in excellent agreement and allay thermal heating concerns raised by prior studies when activating a wide variety of photoactivatable compounds and biological species. We have designed and implemented two plug and play light activated systems for both the Vitrobot Mark IV and the Leica EM GP2 that provide capabilities for light activated cryoTREM studies in the millisecond to minute regime suitable for a wide range of investigations. The complete component list for assembling both systems is provided in order to allow other investigators to assemble similar systems at low cost. The results presented should enable other investigators to readily implement cryoTREM within the standard workflow of cryoEM.

## Methods

Theoretical consideration of heating. The penetration depth for various metals and energies was calculated using a uniform x-polarized plane wave $\bar{E\left(z\right)}=\hat{x}E_{i}e^{-ik_{a}z}$ normally incident upon the smooth surface of the grid. Here $E_{i}$ is the amplitude of the electric field (V/m), $k_{a}$ is the wave number in the incident medium (air), and *i* ^2^ = −1. This plane wave induces conduction currents near the surface (*z = 0*) of the copper or gold grid. The superposition of the incident wave and the reflected wave above the surface can be represented as

$$\bar{E\left(z<0\right)}=\hat{x}E_{i}e^{-ik_{a}z}+\hat{x}E_{r}e^{ik_{a}z}$$ (1)

$$\bar{H\left(z<0\right)}=\left(\hat{y}E_{i}e^{-ik_{a}z}-\hat{y}E_{r}e^{ik_{a}z}\right)/\eta_{0}$$ (2)

where $\eta_{0}=\sqrt{\frac{\mu_{0}}{\varepsilon_{0}}}$ is the impedance of the incident medium, $\mu_{0}$ is the permeability of vacuum, and $\varepsilon_{0}$ is the permittivity of vacuum. For simplicity, we are treating the incident medium as vacuum. $\bar{H}$ is the magnetic field (A/m) found from electric field by Ampere’s law. The transmitted wave in the metallic grid is given by

$$\bar{E\left(z>0\right)}=\hat{x}E_{m}e^{-ik_{m}z}$$ (3)

$$\bar{H\left(z>0\right)}=\left(\hat{y}E_{m}e^{-ik_{m}z}\right)/\eta_{m}$$ (4)

where $\eta_{m}$ is the impedance of the metallic medium, $E_{m}$ is the electric field amplitude of the transmitted wave, $k_{m}=\omega \sqrt{\mu_{m}\varepsilon_{m}}$ and is the wave number in metal, and $\mu_{m}$ and $\varepsilon_{m}$ are the permeability and the permittivity, respectively, of the metal.

The continuity of tangential components of electric fields at the surface yields

$$E_{i}+E_{r}=E_{m}=>1+\Gamma =T$$ (5)

where $\Gamma =\frac{E_{r}}{E_{i}}$ is the complex reflection coefficient, and $T=\frac{E_{m}}{E_{i}}$ is the complex transmission coefficient.

In the absence of a pure surface current, the tangential components of the magnetic fields are continuous

$${\left.\hat{z}\times \left[\bar{H\left(z<0\right)}-\bar{H\left(z>0\right)}\right]\right|}_{z=0}=0$$

$$=>\frac{E_{i}-E_{r}}{\eta_{0}}-\frac{E_{m}}{\eta_{m}}=0$$ (6)

However, the incident wave induces current ${\bar{J}}_{c}$ (parallel to the surface which is considered ideally smooth and lie on the *xy* plane) on the metallic grid. The amplitude of this current decays exponentially as the distance from the surface increases, and this decay is defined by the imaginary part of the wave number. The total conduction current can be treated as an equivalent surface conduction current

$$\bar{J_{sc}}=\int_{0}^{\infty }{\bar{J}}_{c}\left(z\right)dz$$

where $\bar{J_{sc}}$ has the unit of A/m. Using the relation ${\bar{J}}_{c}\left(z\right)=\sigma {\bar{E}}_{m}\left(z\right)$ in the above equation

$$\bar{J_{sc}}=\sigma \int_{0}^{\infty }{\bar{E}}_{m}\left(z\right)dz=\sigma \int_{0}^{\infty }{\bar{E}}_{m}e^{-z/D_{p}}dz=\sigma D_{p}{\bar{E}}_{m}$$ (7)

Here $D_{p}$ is the wave penetration depth, and σ is the complex conductivity. Now, using

$$\left|\hat{z}\times {\bar{\left.H\left(z>0\right)\right|}}_{z=0}\right|=\left|\bar{J_{sc}}\right|$$

in Eq. 6 we get

$$1-\Gamma =\sigma D_{p}\eta_{0}T=\sigma D_{p}\eta_{0}\left(1+\Gamma \right)$$

$$=>\Gamma =\frac{1-{{\sigma D}_{p}\eta }_{0}}{1+{{\sigma D}_{p}\eta }_{0}}$$ (8)

The reflectivity is given by ${\left|\Gamma \right|}^{2}$ , and the fraction of dissipated power is

$${1-\left|\Gamma \right|}^{2}$$ (9)

The dielectric function for metal in the optical frequency range is given by $\varepsilon =\varepsilon_{0}\varepsilon_{r}+i\varepsilon_{0}\varepsilon_{i}$ , where $\varepsilon_{r}$ is the real part and $\varepsilon_{i}$ is the imaginary part of the material. The real part consists of contributions from free electrons as well as bound electrons. But the imaginary part is due to essentially a single source depending on whether the frequency is above or below that corresponding to the interband (*d* to *s*) transitions (Ehrenreich and Philipp, 1962). Above this frequency the dielectric function can be expressed as

$$\varepsilon =\varepsilon_{0}\left(1-\frac{{\omega_{p}}^{2}}{\omega^{2}}\right)+\varepsilon_{0}\varepsilon_{r}^{b}+i\varepsilon_{0}\varepsilon_{i}^{b}$$

where $\varepsilon_{r}^{b}$ and $\varepsilon_{i}^{b}$ are the real and imaginary parts due to bound electrons. Free-electron contribution is negative below the plasma frequency and signifying reflection from the medium. The contribution due to bound electrons is positive which results in bringing the real part closer to zero. When the total real part is very close to zero or positive, the wave is strongly absorbed.

The complex wavenumber is given by $k=\frac{\omega }{c}\sqrt{\varepsilon_{r}+i\varepsilon_{i}}=k_{r}+k_{i}$ . The magnitude of the imaginary part, $k_{i}$ , determines the rate at which the fields attenuate in metal, and its reciprocal is defined as the penetration depth *D*_*p*_. Similarly, the conductivity of a metal in the optical frequencies is a complex function, given by

$$\sigma =i\omega \varepsilon_{0}\left(1-\varepsilon_{r}-i\varepsilon_{i}\right)=\omega \varepsilon_{0}\varepsilon_{i}+i\omega \varepsilon_{0}\left(1-\varepsilon_{r}\right)$$

We observe that the complex conductivity is a function of frequency and various dielectric components. The overall effect is a combination of the dissipative (real) part, different from the DC conductivity value, and the reactive (imaginary) part. For copper at 365 nm, the conductivity is $\left(2.06+i1.37\right)\times 10^{5}$ S/m compared to the DC value of $5.8\times 10^{7}$ S/m. The real part of conductivity is two orders of magnitude lower in the UV region. The complex conductivity and the penetration depth are calculated using the dielectric function. Finally, the absorptivity of the material can be estimated from Eqs. 8, 9.

Power calculations for Table 1. Assuming that the non-linear effects due the absorption of photoproducts are negligible during the early short period of the reaction, the required power that the source must deliver at the grid can be calculated by the following:

$$P=\frac{ChNVc_{p}}{\lambda t\varphi \left(1-10^{-\varepsilon cL}\right)A_{l}}$$

where P is the required power at the grid, W/cm^2^, *C* is the speed of light (m/s), *h* is Plancks constant (J.s), *N* is Avogadro’s number, *V* is the liquid volume (L), *c*_*p*_ is the desired photo product concentration (M), *λ* is the excitation wavelength (m), *t* is the exposure time (s), *φ* is the quantum yield of the photoreaction, *ε* is molar absorptivity (M^-1^cm^-1^), *c* is the starting material concentration (M), *L* is the absorption path length or liquid depth (m) and *A*_*l*_ is the liquid surface area (cm^2^). Assuming that the light delivery system is installed so that the sample side of the grid is illuminated, the area of light absorption is 0.07068 cm^2^, and the liquid depth is 100 × 10^–7^ cm and the volume of liquid is 7.07 × 10^–10^ L.

Integrated controller design. The operation of the light trigger system is controlled by an ATMEGA 328P based microcontroller board (Arduino Nano). This board is programmed to receive some basic parameters from the user and execute the timed signal generation for turning the LED on and off. The board is connected to a 2.8” 320 × 240 pixel touch screen to interact with the user as well as to get basic commands and parameters. Two-way communication between the microcontroller and the display is accomplished through serial peripheral interface (SPI) protocol. The microcontroller sends appropriate command signals to the digital-to-analog converter to generate timed voltage for the LED driver. This voltage is proportional to the LED output power. The LED driver channels a relatively high driving voltage of 24 V to the LED. To synchronize the LED activation with the procedures in Vitrobot a signal from the foot pedal is directed to one of the input ports of the microcontroller. The specific input port is interrupt capable so that the dependent processor operations are done in a precisely timed manner. An example scenario could be turning the LED on after a user supplied delay which may be roughly the blotting time. The controller starts a timer as soon as the user presses the foot pedal to start blotting. After the specified delay has been completed, the controller sends signals for LED activation. This design of open input ports with interrupt capabilities is quite simple yet universal in that synchronous signals from any time-point within the sample preparation as well as any other kind of sensor can be integrated to this system. This makes the controller suitable for installing onto other vitrification instruments with ease. In the Vitrobot application the exposure time was determined to be 17 ms and the delay between exposure and liquid ethane bath was 54 ms. The implementation on the Leica EM GP2 system provides longer exposure times and variable delays.

Beam profile determination. The output beam profile was characterized using a linear photodiode array (TSL1406, AMS-OSRAM AG). This device has 768 photodiodes each of 63.5 µm by 55.5 µm. The sensitivity of each pixel at 365 nm is 11.25 V/(µJ/cm^2^), and minimum integration time is 66 µs. It is noted that a good sensitivity in the UV range is critical for selecting the sensor in beam profile measurement. We found the sensitivity to be more than enough so that we had to use a combination of neutral density filters to prevent pixel saturation even when the LED was driven by current near the threshold value. The sensor was mounted on a linear stage having a translational resolution of 10 µm. Since in our system the approximate beam diameter beyond the condenser lens is 16 mm, there were a maximum of 252 pixels covered by the UV beam. This implies that there were enough pixels to capture a smooth intensity variation along the sensor array. The liquid light-guide (LLG) and lens assembly was kept at a fixed position with respect to the laboratory coordinate system and was aligned so that the beam center is overlapped with the central segment of the sensor array. Pixel data were acquired by a high-speed microcontroller (Teensy 3.6 Board, 180 MHz) through analog ports, and subsequently transferred to the host desktop computer through USB connection. Beam intensity was recorded by aligning the pixel array along the center of the beam and subsequent intensities were collected by translating the linear stage, along with the sensor, by a known distance in the direction perpendicular to the pixel array. Pixel data were post-processed by correcting for the flat-field as well as by normalizing the intensities to provide the two dimensional intensity profile of the beam and the contour map. The intensity profile closely resembles that of a Gaussian beam and moreover, the central area of 2-mm diameter corresponding to the effective EM-grid area contains the dominant uniform intensity. The intensity within this area varies from 80% of the maximum value near the periphery to 100% at the central irradiation zone.

Grid exposure timing measurement. Grid exposure times and energy deposition were determined using a hall sensor First, we measured the beam diameter (d_B_) projected on a plane orthogonal to the radiation propagation and containing straight line of grid travel. The goal was to determine the time the freely falling grid takes to pass this distance provided the path segment is vertically positioned exactly where the beam is located. We used two small magnets (1.59 mm × 1.59 mm × 1.59 mm Neodymium, B111 K&J Magnetics) and a hall-effect latch sensor (DRV5011, Texas Instruments Inc.) to get the timing signals. We configured the setup where the two magnets were mounted on the tweezers. One at the tip of the tweezer and the other separated by a distance of d_B_ corresponding to the grid beam diameter. The hall sensor was mounted on the fixed supporting rod extending beyond the chamber through the sample loading port. The sensor was fixed at the upper most position of the UV beam. As the plunger falls after the blotting operation, the hall sensor output signal changes levels (high: 5 V, low: 0 V) twice due to the two magnets. These changes were captured using an oscilloscope. Since the typical response time of the sensor is 13 µs, this method results in an accurate estimate of the grid exposure duration of 17 ms while falling toward liquid ethane. It is noted that this exposure time can also be calculated from the fundamental equations of Newtonian mechanics considering free fall under gravity.

**FIGURE 3:**
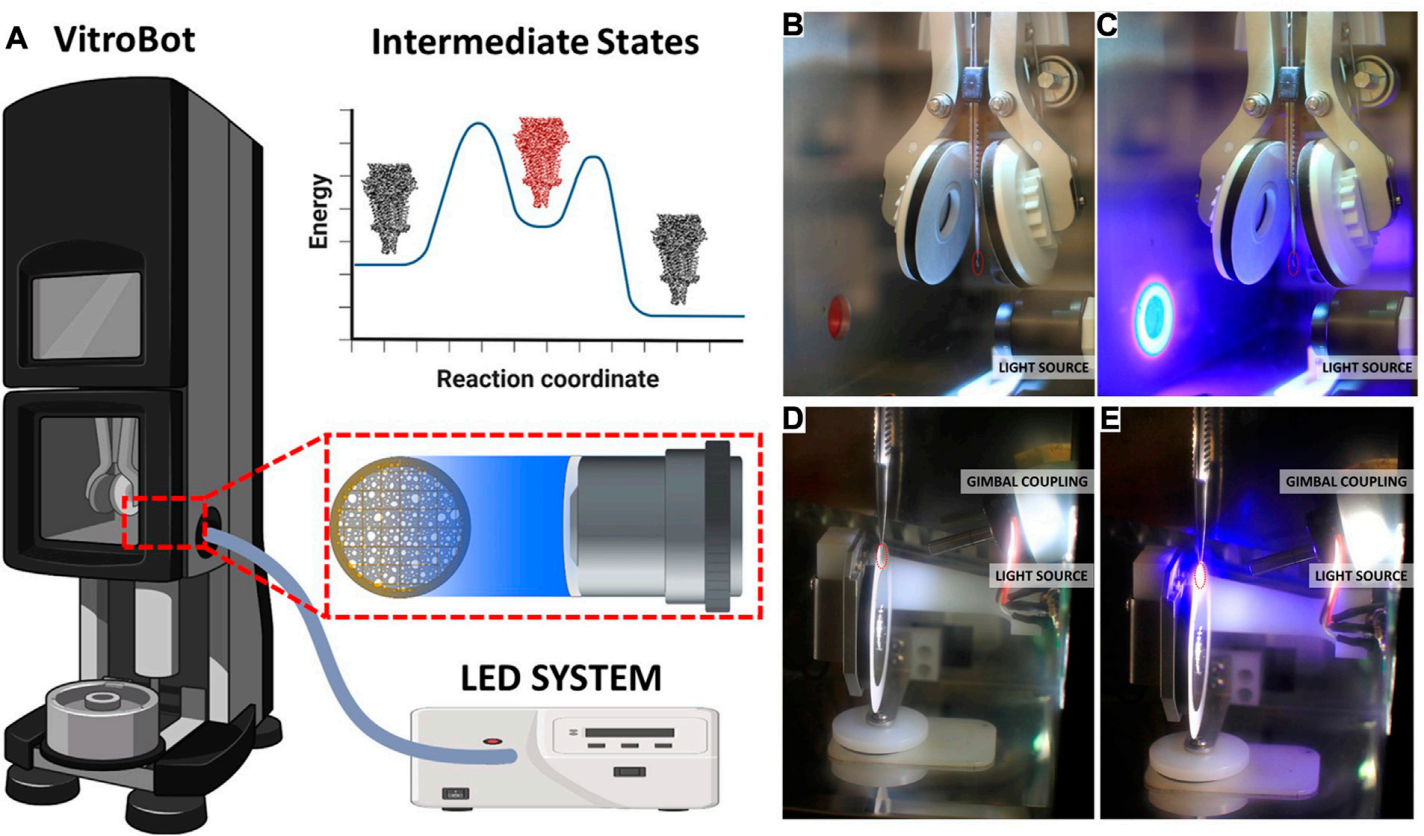
LED system with liquid light guide for plug and play cryoTREM. **(A)** Schematic overview of the system as implemented on the Vitrobot Mark IV, showing the LLG and lens location. A reaction coordinate illustrating the target trapping of an intermediate state (red). **(B)** Photograph of the Vitrobot environmental chamber with the focusing optics unit installed. **(C)** Photograph of the Vitrobot environmental chamber with the focusing optics unit installed and the 365 LED light on. **(D)** Photograph of the GP2 environmental chamber with the focusing optics and gimbal system installed. **(E)** Photograph of the GP2 environmental chamber with the focusing optics and gimbal system installed and the 365 LED light on. The red circles indicate the location of the EM grid prior to blotting. See Supplemental Material for videos of the system in operation.

Optical Train Design. Components for the Vitrobot and GP2 are listed in Table 4. The high-power UV LED (LZ4-V4UV0R-0000, 5.7 W, Osram Sylvania Inc.) is mounted on a metal core printed circuit board (MCPCB) which in turn is mounted on a heatsink. We mounted the LED on the heatsink for the ease of assembly. But the heatsink also provides an additional safety in case of prolonged LED operation. The input end of the liquid-light guide is carefully assembled with the LED-heat sink part such that the core of the light guide is centered with that of the LED. In addition, the distance of the light guide from the LED surface is minimized to ensure maximum collection of light into the guide. The output end of the light guide is connected to a condenser lens housing, and positioned with respect to the lens so achieve nearly a collimated beam. This lens housing is installed inside the climate chamber of Vitrobot Mark IV, and the lens axis aligns with the center of the grid at the blotting position. For the Leica EM GP2 system, the blotting position of the grid is not at the same vertical position as the sample loading port, which is true in Vitrobot Mark IV system (Figure 3). We designed special adapter assembly for the GP2 system so that the output light beam aims at the grid. The assembly consists of a 3D printed part, a kinematic pitch/yaw adapter and an aspheric condenser lens. The kinematic adapter is used to fine tune the beam direction to correct for any misalignment originating from the inaccuracies of part dimensions and of assemblies. In contrast with the Vitrobot system, where the grid exposure to the light beam is fixed, the GP2 system provides some flexibility in varying the exposure time. This flexibility comes from the fact that the retraction of the blotting arm requires approximately one second before the plunger falls toward the liquid ethane. However, the duration of blotting process may vary depending on the user’s preference and sample type. Light exposure is performed only after the blotting process has been completed to achieve accurate, reproducible and predictable photoproduct concentration. Therefore, the detection of the beginning of blotting process can be utilized to accurately time the light exposure. We designed a wall-mounted photointerrupter based sensor that detects the beginning of the blotting process by sensing the change in the reflected light caused by the blotting arm. The timer in the microcontroller starts after being triggered by the sensor, and the instant of turning the LED on is calculated as follows:

$$LED\;Trigger\;time\;\left(T_{Trig}\right)=T_{0}+T_{B}+T_{c}-T_{LED}$$

Where $T_{Trig}$ is the time at which the LED is activated, $T_{0}$ is the time at which the photointerrupter is activated, $T_{B}$ is the blot time, $T_{c}$ is a constant delay fixed within the Leica EM GP2 program following blotting, and $T_{LED}$ is the exposure time form the LED for photoactivation or photoconversion.

**TABLE 4:** Optical train components.

Part	Vitrobot Mark IV	Leica EM GP2
Arduino Nano board (Arduino.cc)	✓	✓
Digital-to-analog converter (MCP4822, Microchip Inc.)	✓	✓
LED driver (RCDE-48–1.05, Recom Power GmbH)	✓	✓
ILI9341 2.8″SPI TFT LCD Display (Amazon.com)	✓	✓
Bus Transceiver (SN74LVC245ANE4, Texas Instruments Inc.)	✓	✓
Photointerrupter (RPR-220, ROHM Co.)		✓
Kinematic Pitch/Yaw Adapter (KAD11NT, THORLABS Inc.)		✓
3D Printed Wall adapter (Fits sample loading port, Custom designed, *stl* files in supplementary)	✓	✓
Liquid Light-guide (805–00038, Excelitas Technologies Corp.)	✓	✓
6 V DC power supply (Mouser Electronics Inc.)	✓	✓
24 V DC power supply (Mouser Electronics Inc.)	✓	✓
ABS Plastic Enclosure (B075WZRW75, Amazon.com)	✓	✓
Aspheric Lens (ACL12708U-A, THORLABS Inc.)	✓	✓
Threaded Adapter (AD9F, THORLABS Inc.)	✓	
Lens Tube (SM1M10, THORLABS Inc.)	✓	
External thread to optic adapter (SM05P05, THORLABS Inc.)	✓	
Adapter with external and internal thread (SM1A6, THORLABS Inc.)	✓	
LED (365 nm, LZ4-44UV00-0000, AMS OSRAM AG)	✓	✓
LED mount and heat sink (M375L4, THORLABS Inc.)	✓	✓
Threaded adapter (AD12F, THORLABS Inc.)	✓	✓
Lens tube (SM1L10, THORLABS Inc.)	✓	✓
Cooling fan (CFM-5010V-052–300, Mouser Electronics Inc.)	✓	✓

Temperature measurements. We used a fine-gage J-type thermocouple (IRCO-001, Omega Engineering Inc.) to measure the temperature change in the grid due to irradiation with 365 nm. The schematic of the experimental setup is shown in Figure 2 and Supplementary Figure S1. There are two major factors in the temperature sensing of the grid that need to be considered. First, the sensor itself must be minimally affected by the radiation. This is related to the mechanism of sensing and/or the arrangement of the sensor. When subject to an intense blue light or UV radiation, the thermocouple typically gives unrealistic readings originating from the photothermal effects. We also experimented with thermopile-based infrared sensor to sense the grid temperature. This type of sensor could not be reliably used due to the absorption of the built in filters, the uncertainty with the emissivity values of grids, and the mismatch between the field-of-view and the grid dimension. The second factor is that the sensor must not thermally load the grid significantly, which is related to the dimensions and hence, the time constant of the sensor. The thermocouple used here is of iron-constantan with approximately 25 micron bead. We mounted the stainless steel tweezer holding the grid on a 3-axis manipulator to adjust the position of the grid relative to the sensor bead. After holding a new grid by the tweezer and mounting on the manipulator, the grid position is lowered such that the bead touches the bottom surface of the rim segment of the grid. Next, the copper mask is positioned so as to minimize the exposure of the thermocouple leads to the light beam. Finally, the output end of the light guide is positioned so that the central portion of the light beam hits the top surface of the grid. Thus, we minimized any spurious reading from the thermocouple by keeping the bead and the leads away from the light beam.

Finite-element simulation. To gain better insight of the grid temperature rise due optical stimulation a finite-element based simulation model was developed in COMSOL Multiphysics. The geometry is developed to represent a 25 µm thick 300-mesh grid held by a stainless-steel tweezer. The model solved the three-dimensional time-dependent heat conduction equation:

$$\nabla^{2}T=\frac{1}{\alpha_{T}}\frac{\partial T}{\partial t}$$

where, *T* is the temperature, *α*_*T*_ is the thermal diffusivity, $\nabla^{2}$ is the Laplacian operator. The top surface of the grid model is specified as the recipient boundary of heat flux which is found from the irradiance multiplied by the absorptivity of copper or gold. Since we are modelling the short early periods of light activation, convective heat transfer to the surrounding air and radiative heat transfer to surrounding objects are not included considering their insignificant percentages. Assuming the convective heat transfer coefficient of 27 W/°C-m^2^ for air, the rate of convective loss is estimated to be 1.9 mW when the grid is at 10°C above the room temperature. The grid absorbs at a rate of 107.4 mW of the 365 nm beam in our system. The rate of radiative loss is estimated to be 25 µW. However, conductive heat transfer to the tweezer is included in the model.

## Data Availability

The raw data supporting the conclusion of this article will be made available by the authors, without undue reservation.
