# Supplementary material for: A simple flash and freeze system for cryogenic time-resolved electron microscopy
Source: Front Mol Biosci. 2023 Mar 7;10:1129225. doi: 10.3389/fmolb.2023.1129225 (PMC10028177; doi:10.3389/fmolb.2023.1129225)
Supplement: Supplementary file 1 [file DataSheet1.docx]

Supplementary Material

Article Title

**Biddut Bhattacharjee*, Md Mahfuzur Rahman, Ryan E. Hibbs and Michael H. B. Stowell***

**Correspondence:** Corresponding Authors: [biddut.bhattacharjee@colorado.edu](mailto:biddut.bhattacharjee@colorado.edu) or [stowellm@colorado.edu](mailto:stowellm@colorado.edu)

## Supplementary Web Accessible Data.

## The controller code, STL files as well as an operations manual are freely available on GitHub at https://github.com/Stowell-Lab/photoflashV1.

## Supplementary Figures and Videos


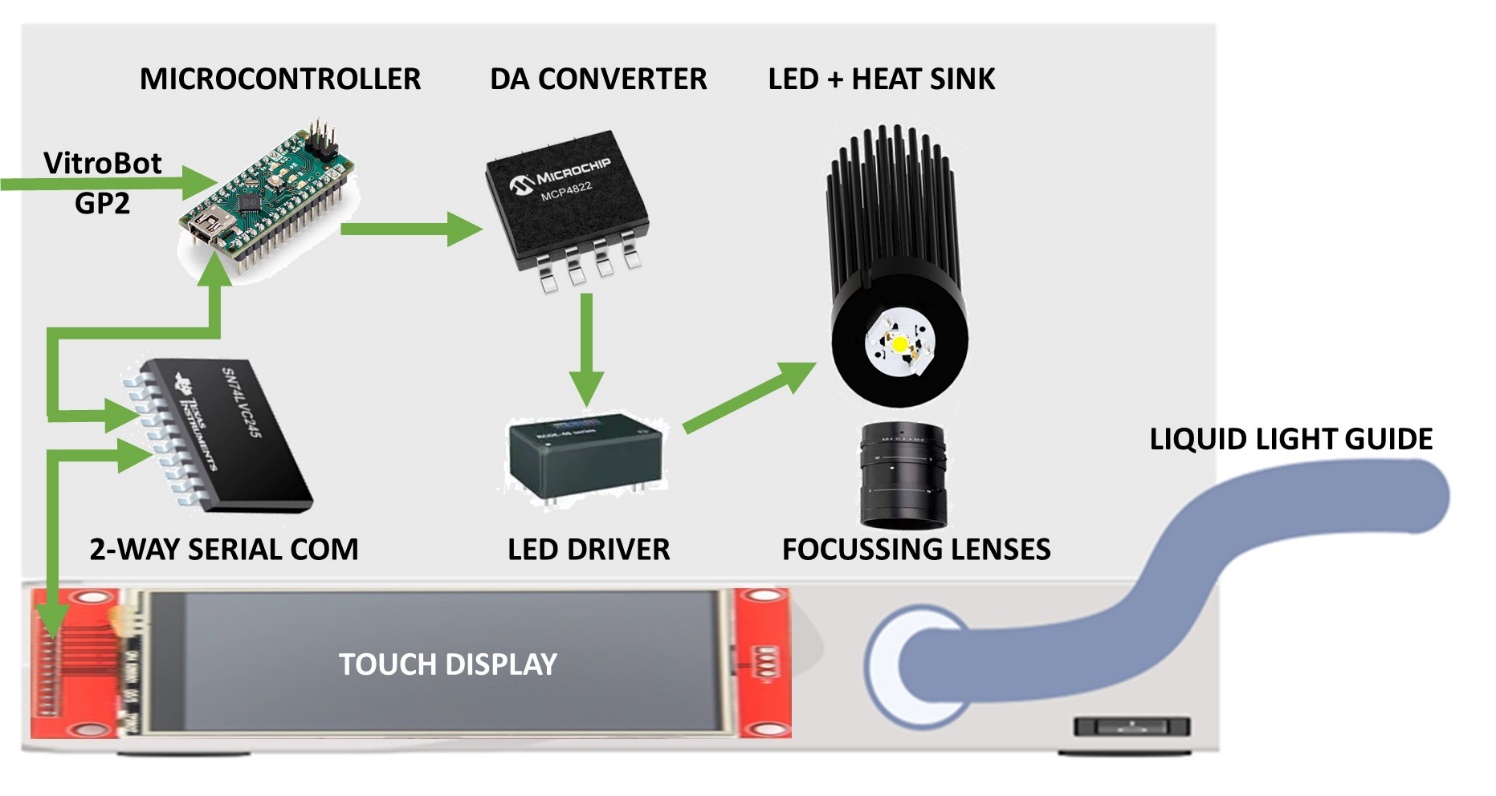


**Supplementary Figure 1.** Schematic and components of the LED control electronics. Refer to Table 4 for manufacturing part numbers.


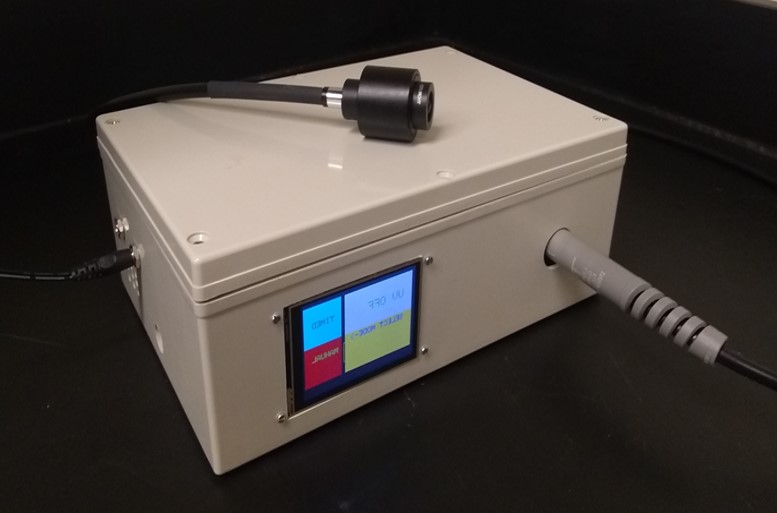


**Supplementary Figure 2.** Photograph of the fully assembled LED system**.**

**Supplementary Video 1.** Video of VitroBot Mark IV in operation with the LED light source**.**

**Supplementary Video 2.** Video of GP2 in operation with the LED light source.
